# Barriers to and facilitators of implementing colorectal cancer screening evidence-based interventions in federally qualified health centers: a qualitative study

**DOI:** 10.1186/s12913-024-11163-0

**Published:** 2024-07-10

**Authors:** Emanuelle M. Dias, Joe R. Padilla, Paula M. Cuccaro, Timothy J. Walker, Bijal A. Balasubramanian, Lara S. Savas, Melissa A. Valerio-Shewmaker, Roshanda S. Chenier, Maria E. Fernandez

**Affiliations:** 1 UTHealth Houston School of Public Health, Houston, TX USA; 2 UTHealth Houston Institute for Implementation Science, UTHealth Houston School of Public Health, Houston, TX USA

**Keywords:** Barriers, Cancer, CFIR, Colorectal cancer screening, Content analysis, Evidence-based interventions, Facilitators, Implementation, Oncology

## Abstract

**Background:**

There is an urgent need to increase colorectal cancer screening (CRCS) uptake in Texas federally qualified health centers (FQHCs), which serve a predominantly vulnerable population with high demands. Empirical support exists for evidence-based interventions (EBIs) that are proven to increase CRCS; however, as with screening, their use remains low in FQHCs. This study aimed to identify barriers to and facilitators of implementing colorectal cancer screening (CRCS) evidence-based interventions (EBIs) in federally qualified health centers (FQHCs), guided by the Consolidated Framework for Implementation Research (CFIR).

**Methods:**

We recruited employees involved in implementing CRCS EBIs (e.g., physicians) using data from a CDC-funded program to increase the CRCS in Texas FQHCs. Through 23 group interviews, we explored experiences with practice change, CRCS promotion and quality improvement initiatives, organizational readiness, the impact of COVID-19, and the use of CRCS EBIs (e.g., provider reminders). We used directed content analysis with CFIR constructs to identify the critical facilitators and barriers.

**Results:**

The analysis revealed six primary CFIR constructs that influence implementation: information technology infrastructure, innovation design, work infrastructure, performance measurement pressure, assessing needs, and available resources. Based on experiences with four recommended EBIs, participants described barriers, including data limitations of electronic health records and the design of reminder alerts targeted at deliverers and recipients of patient or provider reminders. Implementation facilitators include incentivized processes to increase provider assessment and feedback, existing clinic processes (e.g., screening referrals), and available resources to address patient needs (e.g., transportation). Staff buy-in emerged as an implementation facilitator, fostering a conducive environment for change within clinics.

**Conclusions:**

Using CFIR, we identified barriers, such as the burden of technology infrastructure, and facilitators, such as staff buy-in. The results, which enhance our understanding of CRCS EBI implementation in FQHCs, provide insights into designing nuanced, practical implementation strategies to improve cancer control in a critical setting.

**Supplementary Information:**

The online version contains supplementary material available at 10.1186/s12913-024-11163-0.

## Background

Although colorectal cancer screening (CRCS) can increase the early detection of cancer, it remains underutilized, particularly in underserved populations [1]. The U.S. Preventive Services Task Force recommends CRCS for average-risk individuals aged 45–75 years [2]. In 2021, the average rate of CRCS for adults in the United States was 58.7%, and the task force’s goal is to increase the rate to 68.3% [3]. The COVID-19 pandemic contributed to a decrease in CRCS of about 25% below baseline levels compared to the start of the pandemic [4]. Further, there are significant gaps across the United States, and Texas currently ranks 48th in screening nationwide, with a current average rate of 59.6% [5, 6].

Federally qualified health centers (FQHCs) are community-based health centers that provide comprehensive cancer screenings to medically underserved populations [7]. In 2022, 90% of FQHC patients fell below the poverty level, and 20% were uninsured [8]. In Texas, the average CRCS rate among FQHC patients is 35% [9]. Because this falls well below national screening goals, there is an urgent need to increase CRCS uptake in Texas FQHCs. Further, given the CRCS reporting requirements that tie incentive payments to quality measures, there is motivation for FQHCs to increase CRCS rates [10].

There is empirical support for the ability of evidence-based interventions (EBIs) to increase CRCS across various settings. Notably, *The Guide to Community Preventive Services (i.e., Community Guide)* provides recommendations about EBIs based on systematic reviews of studies on these interventions [11, 12]. Despite support for EBIs, the use and implementation of CRCS in FQHCs remains low, particularly compared to other integrated health systems, which may be due to organizational factors (e.g., staff turnover) [13–15]. To increase the use of EBIs in the FQHC setting and the uptake of CRCS, it is crucial to identify any barriers and facilitators associated with implementing EBIs relevant to CRCS.

This study draws upon the Consolidated Framework for Implementation Research (CFIR) [16] and organizational readiness, supported by the Interactive Systems Framework (ISF) and represented by the R = MC^2^ (Readiness = Motivation x Innovation-Specific Capacity x General Capacity) heuristic [17]. CFIR is a widely used determinants framework that guides users when investigating barriers and facilitators through its constructs across five domains that influence implementation [18, 19]. In 2022, the CFIR was expanded to encompass equity-related domains and constructs. The updated CFIR comprises 48 theory-based constructs and is organized into five domains of context, where users can systematically assess CFIR constructs throughout several domains (e.g., work infrastructure from the inner setting domain) [16]. ISF guides the implementation of innovations, and organizational readiness is a key element of the process. R = MC^2^ is used to define what it means to be ready [17].

Although some research has focused on determinants of CRCS [20–23], no known studies use the updated CFIR to explore barriers to and facilitators of CRCS EBIs within FQHCs. Using CFIR to analyze semi-structured interviews can help to determine the barriers to and facilitators of the implementation of CRCS EBIs in the FQHC setting. Thus, this study used a qualitative analysis guided by CFIR to identify these barriers and facilitators in FQHCs.

## Methods

### Parent study

The parent study, funded by the Centers for Disease Control and Prevention (CDC) Colorectal Cancer Control Program (CRCCP), aimed to increase CRCS among populations served by Texas FQHCs. The program’s intent is to expand the use of *Community Guide-*recommended EBIs, which include provider or patient reminders, provider assessment and feedback, and strategies to reduce structural barriers to increase CRCS among individuals aged 45–75 years [24, 25].

Provider reminders are alerts integrated into electronic health records (EHR) that inform healthcare providers of a patient due for CRCS. Patient reminders are often written correspondence (e.g., letters) or telephone messages alerting them that they are due for a screening. Provider assessment and feedback refers to interventions that evaluate a provider’s performance in delivering screening. Reducing structural barriers refers to approaches or strategies that ease non-economic burdens that can make accessing cancer screening difficult [24]. Program activities to increase priority EBIs include formal partnerships with FQHCs, the use of community advisory councils, organizational needs assessments, and strategies to support implementation (e.g., practice facilitation).

### Sampling strategy, context, and researcher characteristics

The parent study team (two research coordinators and a program manager; hereafter, team) purposefully sampled study participants. Before recruitment, the team secured support for the project from each member of FQHCs’ leadership. The team identified champions in four health systems to assist in recruiting individuals whose roles are needed for CRCS involvement and in implementing CRCS EBIs at each clinic site. Champions use their organizational knowledge to negotiate administrative barriers within their system to build implementation support [26]. System champions linked potential participants with the team for recruitment and interview scheduling. The Committee for the Protection of Human Subjects at UTHealth Houston approved all study procedures and protocols (HSC-SPH-20-1118).

### Data collection

The team conducted 23 semi-structured group interviews among four FQHC systems in Southeastern Texas from April 2021 to March 2022. The team selected these FQHC systems because of low reported CRCS rates. Due to the COVID-19 pandemic, all interviews were conducted via Zoom. The four trained interviewers co-facilitated the interviews and tested the interview guide to improve the flow and estimate the interview length. All interviews lasted 45 min to an hour and were audio-recorded using Zoom’s recording functionalities. Rev Transcription Services transcribed all audio-recordings. Participating clinics received a check for $1,000 following the completion of the interviews. After collecting all interviews, the team reviewed the transcription accuracy by listening to the recordings.

All group interview participants verbally consented to participate in the study. After each interview, participants completed a brief sociodemographic questionnaire on REDCap. The team conducted two types of interviews: General clinic and evidence-based intervention (EBI). The team developed interview guides informed by the CDC CRCCP assessment tool [25], CFIR [16], and the National Colorectal Cancer Roundtable evaluation toolkit [27]. The general clinic interview asked clinic leadership (e.g., clinical managers and quality directors) about their experiences with practice change, CRCS promotion, quality improvement initiatives, organizational readiness (R = MC^2^ heuristic) [28], and the impact of COVID-19. The EBI interview concerned the use of CRCS EBIs across perspectives of varying clinic staff (e.g., primary care providers, nurses, and medical assistants). The interview guides covered several sections (Additional files 1 and 2).

### Secondary analysis of data

#### Data analysis

This research is a secondary analysis of a subset of semi-structured interviews conducted with FQHC staff by the CDC CRCCP team. We conducted a directed content analysis to identify theoretical patterns within qualitative data [29]. We developed a codebook, using 48 deductive codes based on the updated CFIR constructs and subconstructs [16], and added inductive codes to capture concepts not represented initially. Using the updated CFIR framework was significant in this study, given the focus on innovation recipients (e.g., clinic patients) and the addition of equity-related determinants to the framework that can influence the implementation process. This change is particularly relevant because equity-related determinants are essential for understanding the barriers to and facilitators of implementation in FQHCs that serve medically underserved populations.

We used ATLAS.ti Version 9 and two coders independently read and coded all 23 interview transcripts and compared coding across transcripts. We analyzed codes for patterns, developed summary statements, and selected representative participant quotes. We continued analysis until we achieved thematic saturation [30, 31]. When reporting the findings, we used the Standards for Reporting Qualitative Research [32].

## Results

Table 1 displays participant demographics. There were 59 participants across 23 group interviews, including 2–3 participants in each interview. During the interviews, we identified six CFIR constructs related to the barriers to and facilitators of the EBIs: information technology infrastructure, innovation design, work infrastructure, performance measurement pressure, assessing needs, and available resources. These findings are presented in terms of the definitions of CFIR constructs (Table 2). We determined the most salient concepts based on the frequency of codes and richness of data. The most frequently coded constructs were work infrastructure (*n* = 135), assessing needs (*n* = 103), innovation design (*n* = 68), and information technology infrastructure (*n* = 68). The constructs that we coded the least were materials and equipment (*n* = 35), external pressure (*n* = 9), capability (*n* = 7), and implementation facilitators (*n* = 7). We also discovered emerging concepts related to staff buy-in. Below, we present the relevant constructs and illustrative participant quotes.

**Table 1:** Qualitative interview participant characteristics

	*n*	Percent (%)
**Demographics**		
Age (Mean, SD)	44.20 (11.48)	
Female	53	90.00
**Race**		
Asian, Native Hawaiian, or Other Pacific Islander	6	10.17
Black, African, African-American	8	13.56
White	45	76.27
Hispanic or Latino Ethnicity (of any race)	20	33.90
**Clinic Role**		
Primary Care Provider (Physician, Nurse Practitioner, or Physician Assistant)	13	22.03
Clinical Manager	9	15.25
Medical Assistant or Nurse	14	23.73
Quality Director	14	23.73
Practice or Project Manager	9	15.25
**Total Number of Participants**	**59**	
**Total Number of Group Interviews**	**23**	

**Table 2:** Updated CFIR constructs and definitions [21]

Updated CFIR Construct	CFIR Construct Definition	Corresponding domain
Information technology infrastructure	Technological systems used for communication, electronic documentation, data storage, management, reporting, and analysis.	**Inner setting**: the setting in which the innovation is implemented (e.g., FQHC clinic).
Innovation design	The innovation being well-designed and packaged, including how is it assembled, bundled, and presented.	**Innovation**: referring to the “thing” being implemented (e.g., provider reminder).
Work infrastructure	The organization of tasks and responsibility between individuals and teams, staffing, to support the team’s performance.	**Inner setting**: the setting in which the innovation is implemented (e.g., FQHC clinic).
Performance measurement pressure	Quality metrics or established goals that help drive implementation of the EBI.	**Outer setting**: setting where the inner setting exists and can be comprised of several levels (e.g., FQHC system with many clinics).
Assessing needs	Strategies used to collect information about the priorities, preferences, and needs of people.	**Implementation process**: activities used to implement the innovation/EBI.
Available resources	Resources that are available to implement and deliver the innovation/EBI.	**Inner setting**: the setting in which the innovation is implemented [e.g., FQHC clinic])

### Information technology infrastructure and innovation design

The CFIR constructs of information technology infrastructure and innovation design were both barriers and facilitators in regard to the implementation of provider and patient reminders.

Participants noted that implementing reminders would require clinics’ EHRs. Most participants demonstrated an understanding of how to use EHRs to execute provider reminders, including the ability to identify patients’ previous screening and whether they were overdue for screening, as indicated through a flag or alert. Nevertheless, participants shared the limitations of using EHRs in their clinics, including the shortcomings of the available data, and noted the need for optimizing current systems and having designated staff to coordinate the systems’ use. A clinical manager stated:

*One of the things that I had issues with is how you [the quality team] pulls data. It means nothing to me to tell me the number of people I miss. Tell me who [is missing a screening test] is so we can go back and order the [screening] test.*

Participants shared other limitations of their current technology, including the design of the alerts or flags on the EHR. Many individuals from different clinics shared that these limitations led to the staff’s feeling overwhelmed and not wanting to use the current technology infrastructure to use the reminders. As noted by a quality director:

*We have [patients’] medical history. But the clinical person needs to watch out for that global alert. They need to intentionally look to where that pulls up. And so, I don’t tend to use those.*

A nurse practitioner said: “There’s not a big flashing warning saying, ‘Hey, the patient hasn’t had colorectal cancer screening.’”.

Participants also discussed how using the EHRs can be helpful. They described EHRs as part of their daily clinical routines (e.g., pre-patient planning), including during morning “huddles,” to remind providers with printouts that designated which patients with appointments were also due for screening. All participants noted the opportunity to use EHR-integrated provider reminders and other reminders during face-to-face interactions or through email.

Similar to provider reminders, the constructs relevant to patient reminders were information technology infrastructure and innovation design. Participants described the staff’s reliance on technology (e.g., EHRs) and optimizations to roll out reminders, using automated features, to patients regarding upcoming appointments and screenings. Participants also described text message, telephone, and email systems that identify patients with overdue screenings and track communications.

Participants also noted the burden that electronic patient reminders put on the team members, as they have to set up systems to implement reminders, and on patients to take action to receive reminders; patients need to “opt in” to receive reminders. As a primary care provider explained:

*Our automated list isn’t giant just because you have to sign up for the text messages or the automatic reminders, and some people don’t do that.*

Clinic staff explained the design process for patient reminders, shared the automated features of EHRs, and explained their support roles in implementing the EBI. For example, if patients do not answer automated reminders, these staff members follow up with the patient personally. A practice manager stated:

*If you’re talking about [screening] appointment reminders, we have an automatic reminder system that contacts the patient 48 h in advance. And, of course, and if they don’t pick up or answer, then we, the front desk, will call the patient in 24 h in advance to ensure they’re coming in.*

Although participants portrayed the complexities of using technology, they also described how technology accelerated the implementation of patient reminders. They spoke about the extensive systems used to reach the populations they serve, such as those that involved several reminders.

### Work infrastructure and performance easurement pressure

Work infrastructure and performance measurement pressure are aligned with provider assessment and feedback. Participants delineated systems/infrastructure already in place to support provider metric monitoring. They used already-established quarterly or monthly meetings to discuss feedback with providers. One quality director described how huddle communication is structured: “And in our huddle teams, our nurses and providers will go over the patients for the next day and they will decide if we need to ask this person or that person for the status on their colorectal screenings.” Other team members described their current provider assessment structure as an opportunity to assign patients to a particular provider, ensuring screening recommendations and facilitating discussions about potential barriers. A quality director described the provider assessment structure of their clinic:

*Whenever we have our provider meetings, we’ll discuss with them [providers]. What roadblocks are you hitting or pushback or where are you having some issues with this? And then we can go over, well, these are the patients you saw, how many qualified for this particular [screening] measure, and how many you completed. Is it a timing issue? Are you just not offering it a lot? So, what must we do to help you get that done?*

Finally, participants portrayed provider assessment and feedback as supported by competitive processes. For example, a clinical manager shared that the clinic was planning to compare provider screening scores through a friendly competition:

*Well, I have a scorecard, a chart in my office. We have discussed trying to do a little competition, but we haven’t figured out how you grade that because some providers have a bigger panel than others. Do you do it on a percentage basis? We’ve talked about that several times, as far as a competition to show them each other’s scores.*

When describing other provider assessment and feedback implementation efforts in the clinic, participants often mentioned FQHC-designated quality measures as facilitators but also shared some difficulties in using data to determine how well clinic providers are performing. A physician assistant noted:

*Since it’s a FQHC, I’m sure they pull those [screening] markers. But I’ve never gotten any feedback on our numbers regarding who’s been referred and who has not offered screening.*

In addition, interviewees shared other assessment strategies actively used in the clinic space to execute provider assessment and feedback. These strategies include monthly chart reviews, provider annual reviews based on quality measures, running and reporting uniform data system measures (i.e., federal reporting requirements for FQHCs), and scorecards of provider success compared to national averages. These assessment strategies relate to the CFIR work infrastructure and performance measurement pressure constructs. According to a physician:

*Once a month, we have a provider meeting, and they present us with a scorecard, which has all of our measures on it, and colorectal cancer screening is one of those. As a clinic, we are given the total number of colon cancer screenings that are done, we’re given the national average. So, it’s compared to those things monthly. Individually, we get a scorecard. It has our numbers on it and will tell us how many people had colon cancer screenings in my cohort, versus how many patients qualified for that particular measure.*

### Needs assessment and available resources

The assessing needs and available resources CFIR constructs were relevant to implementing strategies to reduce structural barriers. Participants noted that case managers, social workers, and referral coordinators assessed patient needs in multiple ways, including reading EHR information, having primary care conversations with patients, or determining program eligibility. Determination of program eligibility involves locating resources or lower-cost types of screening care.

A practice manager described educating patients about available options to reduce the cost of medical services, stating:

*We educate the patients that, if you’re on our sliding fee scale, you’re always welcome to make a payment plan. We don’t put it on any credit report; we put up posters to remind the patients that we’re committed to their care, whether or not they can pay the cost of their medical services.*

These experiences involved similar obstacles to those when serving underserved individuals who had education and language barriers, and there were many “no-shows” and appointment cancellations. Most participants felt that, of those obstacles, transportation was a top barrier. One nurse practitioner stated, “Transportation is a huge barrier for our population, because a lot of our population is just a rural community and leans towards the poorer part of rural.” Another nurse practitioner added:

*I agree that transportation is a big one. We live 30 miles from the closest place that even offers [screening]. And then a lot of our patients live farther out than that. So, to get them to a gastro clinic will be at least an hour drive to and from there, plus all the rigmarole it takes once you get there.*

Clinic team members shared a variety of available resources that clinics offer to reduce structural barriers, including group education sessions, rideshare programs (e.g., Lyft), community health worker navigation, language translation phone lines, bus routes, gas cards to address transportation, and connections with hospitals or other partnerships for certain resource offerings (e.g., screening vouchers). Interviewees detailed many in-clinic funding resources, including an eligibility process to qualify uninsured individuals for reduced-cost care and other programs. As one physician described:

*We put our patients through a process with an eligibility specialist, and they can qualify for one of several grants. Our sliding fee scale can qualify the patients for a co-pay as low as $35 to $75 to full pay, depending on the household size and income.*

### Staff buy-in

Our analysis also identified concepts that did not align with the updated CFIR, including buy-in, a person’s ability to accept or be willing to actively support and participate in implementation. Participants communicated that buy-in was necessary for any implementation success, as it contributes to the overall environment within a setting to enact change. One medical assistant described it as “everybody being on the same page.” Interviewees shared that leadership exemplifies buy-in by endorsing change while providing employees with an explanation of why an organization should roll out a particular initiative. As noted by a clinical manager:

*What is pivotal for the success of any program that gets rolled out or implemented across an organization is having the buy-in from the top to the bottom. Emulating and preaching [are] the change that we want to move forth. So, it’s pivotal to any change.*

A physician added, “So, I think it all ties to buy-in. If you don’t have the climate set for the change you seek, you will be stuck and not have the overall outcomes you’d like. So, it’s all intercorrelated.”

## Discussion

We conducted group interviews with a purposive sample of FQHC staff at four health systems. We found six CFIR constructs (information technology infrastructure, innovation design, work infrastructure, performance measurement pressure, assessing needs, and available resources) regarding implementing four recommended EBIs. We also found emerging concepts that are outside of CFIR. Specifically, we found that the information technology infrastructure (systems used for data communication) and innovation design (how well innovations are designed) of EHR systems used for provider or patient reminders can influence the implementation success in clinics, particularly due to the burden that EHR technology can have on its users.

Our findings are consistent with those in the literature. A recent qualitative study applying the Reach, Effectiveness, Adoption, Implementation, Maintenance (RE-AIM) [33] and CFIR frameworks to examine the implementation planning process noted that technology-based interventions that require an interface with healthcare data systems (e.g., EHR) can be complex to integrate into an organization [34]. The work infrastructure construct relevant to provider assessment and feedback aligns with a similar finding from a qualitative study of the R = MC^2^ heuristic determinants in implementing general and CRCS-related practice changes in FQHCs. The study found that organizational structure (workflow processes) and intra-organizational relationships (relationships within organizations) were relevant to implementation in FQHCs [34].

These findings are consistent with those of other studies that have used the original CFIR to examine the uptake of CRCS and implementation of CRCS EBIs in low-resource healthcare settings. Similar research identifying and describing factors influencing the implementation of evidence-based programs to increase CRCS in five safety-net system sites found that both available resources (e.g., transportation and EHR access) and patient needs and resources (e.g., financial barriers) constructs were salient factors at almost all sites [35]. Lam et al. (2021) aimed to describe factors that hindered or promoted the implementation of three EBIs (i.e., provider reminders, provider assessment and feedback, and patient navigation) to improve CRCS in an urban FQHC. The results of this study align with ours as they also identified the perceived burden among providers using EHR reminders. Although the design of provider reminders linked to EHRs seemed simple, providers became frustrated and ignored the reminder alerts [21].

Soloe and colleagues (2022) interviewed CDC CRCCP partners in various United States locations. This study used CFIR to identify the factors that support readiness to implement EBIs to promote CRCS. They identified information sharing (accessing and sharing patient and EHR data) as supportive of integrated implementation [23]. This finding is consistent with ours, as the information technology infrastructure (i.e., systems used for data communication) that rolls out provider and patient reminders can influence implementation success. In contrast, a longitudinal qualitative case study of four CRCCP awardees partnering with primary care clinics to implement EBIs identified factors related to the original CFIR construct, networks and communication (i.e., the nature and quality of informal and formal communication). Factors contributing to networks and communication include leadership support to establish EBIs and program champions to advocate for sustained implementation among upper clinic administrators [22]. Our study did not identify leadership or high-level leadership as salient constructs, but we identified buy-in ‘from the top’ as an integral implementation facilitator.

Our results, except buy-in, are within CFIR. Buy-in is a highly cited implementation barrier, particularly in clinical and primary care settings [36–39]. Nevertheless, limited literature has characterized buy-in as explicitly linked to CRCS implementation. This concept is also frequently tied to other organizational determinants, such as identifying and training program champions and team-building practices that increase communication between staff [40–42]. Buy-in correlates with the “knowledge and beliefs about the intervention” construct from the original CFIR, defined as the attitudes of individuals toward a particular intervention [43]. The revised CFIR, however, no longer includes this construct, as previous users expressed that it overlapped with several innovation domain constructs [16].

Identifying CFIR and new constructs is pivotal to clinical implementation studies. This study contributes to an understanding of specific barriers (technology infrastructure) and facilitators (available resources) that influence implementation. Our findings can help researchers by informing their selection or design of implementation strategies to improve the use of EBIs in the FQHC context [19]. For example, although the *Community Guide* promotes provider and patient reminders, we found that reliance on EHR and other technology can burden clinic functioning given limited resources and capacity. Instead, research can focus on developing implementation strategies that address other pivotal barriers, such as building staff buy-in for implementation through technical assistance. At the policy level, the study underscores the need for policies that promote technology adoption in FQHCs through grants or incentives.

### Strengths

To the best of our knowledge, this is the first study to use the updated CFIR to explore *Community Guide*-recommended EBIs for CRCS. Notably, the updated framework includes determinants that focus on innovation recipients (e.g., clinic patients). Our study identified information technology infrastructure and assessing needs (newly added constructs) as prominent determinants for our priority EBIs. The newly added performance measurement pressure and work infrastructure subconstructs correspond to larger CFIR constructs. Another strength is the diverse sample, including perspectives from various staff types, ranging from support roles to clinicians and upper-level management.

### Limitations

As with similar studies, this one has its limitations. First, the COVID-19 pandemic presented challenges when conducting interviews. During this time, FQHCs engaged in telemedicine and the vaccination roll-out. In addition, leadership turnover at project FQHCs affected interview recruitment. To incentivize FQHC participation, the team offered technical assistance for implementing CDC CRCCP EBIs. Another limitation is the potential power dynamics in group interviews. We recognize that an inherent hierarchy in clinical settings can shape group interview conversations [44]. Nevertheless, group interviews allow us to address scheduling challenges while minimizing disruptions to clinical services and to be able to offer insight into the implementation process.

## Conclusion

Our study provides a valuable contribution to understanding the determinants of the implementation of CRCS EBIs in FQHCs. We found that the application of CFIR can identify barriers, such as technology infrastructure. This finding underscores the opportunity for expanding grants or other policy incentives that can facilitate the use and adoption of technology in this setting to improve EBI implementation. We also enhance understanding of CFIR constructs within the FQHC setting and identified buy-in as a concept that goes beyond the framework. Our findings offer insight into more nuanced program design and practical implementation strategies to enhance cancer control efforts and improve implementation in a setting critical to improving CRCS in underserved populations.

### Electronic supplementary material

Below is the link to the electronic supplementary material.

Supplementary Material 1

Supplementary Material 2

## Data Availability

The interview data generated and analyzed during the current study are not publicly available to protect the privacy of those interviewed, but are available from the corresponding author on reasonable request.

## Abbreviations

EBI: Evidence-based intervention
FQHC: Federally qualified health center
CRCS: Colorectal cancer screeening
CFIR: Consolidated Framework for Implementation Research
ISF: Interactive Systems Framework
R = MC^2^: Readiness = Motivation x Innovation-Specific Capacity x General Capacity
CDC: Centers for Disease Control and Prevention
CRCCP: Colorectal Cancer Control Program
EHR: Electronic health record
