# Supplementary material for: Barriers to and facilitators of implementing colorectal cancer screening evidence-based interventions in federally qualified health centers: a qualitative study
Source: BMC Health Serv Res. 2024 Jul 10;24:797. doi: 10.1186/s12913-024-11163-0 (PMC11238502; doi:10.1186/s12913-024-11163-0)
Supplement: Supplementary file 1 — Supplementary Material 1 [file 12913_2024_11163_MOESM1_ESM.docx]

**Table 1.** General Clinic Interview Questions

| **Section 1: Examples of Practice Change** |
| --- |
| *Example Question:* Now, I’d like you to take a minute or two to think about an example of a successful practice change within your health center. Something that the health center started doing differently or a new protocol or practice where everything worked well, people were energized and engaged, and things flowed smoothly. Please describe the change and what happened.   - Probe: In your opinion, what made it successful? |
| **Section 2: CRCS at the Health Center** |
| *Example Question:* What does your health center do to promote colorectal cancer screening?   - Probe: Does the clinic use provider or patient reminders? |
| **Section 3: Organizational Readiness** |
| *Example Question:* How do you think a health center’s *leadership* might affect the adoption and implementation of colorectal cancer screening strategies? |
| **Section 4: Quality Improvement Initiatives** |
| *Example Question:* What existing quality improvement initiatives is your clinic working on? |
| **Section 5: COVID-19** |
| *Example Question:* Please describe how COVID-19 has impacted the implementation of the evidence-based approaches for increasing CRCS.   - Probe: Please describe the impact on *patient reminders.* |
