# Supplementary material for: Barriers to and facilitators of implementing colorectal cancer screening evidence-based interventions in federally qualified health centers: a qualitative study
Source: BMC Health Serv Res. 2024 Jul 10;24:797. doi: 10.1186/s12913-024-11163-0 (PMC11238502; doi:10.1186/s12913-024-11163-0)
Supplement: Supplementary file 2 — Supplementary Material 2 [file 12913_2024_11163_MOESM2_ESM.docx]

**Table 2.** Evidence-Based Intervention (EBI) Interview Questions

| **Section 1: Provider Assessment and Feedback** |
| --- |
| *Example Question:* Is there a provider assessment and feedback process currently in place and operational (being used)?   - Probe: Describe who is being assessed (individual providers, clinic teams, clinics). |
| **Section 2: Provider reminder and recall systems** |
| *Example Question:* Is there a provider reminder system (prompts when patient is due to screening) currently being used for colorectal cancer screening?   - Probe: Describe the format (EHR, manual flag or note) and how it is delivered. |
| **Section 3: Patient reminders** |
| *Example Question:* Is there a patient reminder system (patient is told it is time for their screening) that is currently being used for colorectal cancer screening?   - Probe: Describe how patients are being identified and chosen for this EBI. |
| **Section 4: Structural barriers** |
| *Example Question:* Are there currently activities in place and operational (in use) for reducing structural barriers to colorectal cancer screening?   - Probe: Describe how obstacles to screening completion are identified (individual and community needs). |
| **Section 5: Patient navigators** |
| *Example Question:* Does the clinic already use Patient navigation to support CRC screening?   - Probe: Describe the process of the patient navigators. |
